# The Impact of the Instigator Rule on Fighting in the National Hockey League

**DOI:** 10.1155/2022/7024766

**Published:** 2022-02-27

**Authors:** Cole Morrissette, Forrest L. Anderson, Thomas A. Fortney, Liana Tedesco, Venkat Boddapati, Hasani Swindell, David Trofa, Charles A. Popkin

**Affiliations:** Center for Shoulder, Elbow and Sports Medicine, Columbia University Medical Center, New York City, NY, USA

## Abstract

**Background:**

Fighting is often considered an essential part of professional hockey. Increased ticket sales, a means to self-regulate other dangerous gameplay, and helping teams win are a few of the reasons that fighting advocates provide for retaining fighting in the NHL. However, fighting trends have changed over the past 50 years. Given the recent data on concussions and player safety, an in-depth analysis of fighting is required to understand if fighting has a place in the future of the NHL.

**Methods:**

Seasonal statistical team data on NHL teams from the 1967 to 2019 seasons were collected and analyzed using publicly available databases. Specific outcome variables of interest related to fighting, penalties, the final team record for a given season, and final standing were recorded. The data were divided into subgroups according to “era of play” and before/after the implementation of the instigator rule. The trends in fighting, seasonal outcomes, and other minor penalties were assessed to determine the trends in fighting over the past 50 years, the relationship between fighting and winning, and the impact of the instigator rule.

**Results:**

Fights per game decreased significantly after the implementation of the instigator rule (0.71 to 0.51 fights per game, *p* < 0.0001). There was no significant difference in fights per game when comparing Stanley Cup champions to nonplayoff teams in either the modern era (0.36 vs. 0.42, *p* = 0.43) or the expansion era (0.45 vs. 0.51, *p* = 0.49). Only two Stanley Cup champions (the Flyers 1974–1975 and the Ducks 2006–2007) led the league in fighting. A multivariate regression analysis comparing fights per game and points earned per season divided by the number of games played revealed a statistically significant inverse relationship (coefficient = −0.16, *p* < 0.001).

**Conclusion:**

Our analysis demonstrates that the Instigator rule achieved its intended effect to decrease the number of fights per game. In the current era of professional hockey, there is no compelling evidence that a team with more fights per game will achieve greater seasonal success. These results continue to cast doubt on the belief that fighting is a necessary strategy for winning games at the NHL level.

## 1. Introduction

Proponents of fighting in the National Hockey League (NHL) have long considered fighting an essential part of gameplay [1, 2]. While other professional sports leagues have banned fighting altogether and levy immediate ejections or heavy fines, the NHL allows fighting to occur with only a small penalization. The NFL, for example, fines players $36k and $72k for their first and second fighting violations, respectively, while also delivering a 15-yard penalty and an immediate game ejection [3]. As of the 2021–2022 season, the official NHL rules punish participants in a fight with 5-minute matching penalties. If one player is identified as having clearly instigated the fight, that player receives an additional 2-minute penalty and a 10-minute game misconduct. Furthermore, if a player in the NHL is involved in 3 fights in a single game or is found to have instigated 2 fights, they receive a game misconduct. Lastly, any player that leaves the bench to join a fight is also given a game misconduct [4].

The natural history of fighting in the NHL, however, is nuanced and has been modified several times since the inception of the league. In 1922, the NHL officially recognized that fighting would be penalized similarly to other lesser rule violations, enabling a culture of violent gameplay [5,6]. During this time, advocates of fighting believed it could increase a team's chances of winning [7]. This belief has persisted since those early days. The 1975 Philadelphia Flyers, nicknamed the “Broad Street Bullies,” won two consecutive Stanley Cup championships and are often used as evidence of this strategy [6, 8]. Others have provided evidence to refute this notion [9]. Proponents of fighting often identify other reasons for fighting including self-regulating dangerous gameplay, changing intragame momentum, and ticket sales [6, 9, 10].

**Figure 1:**
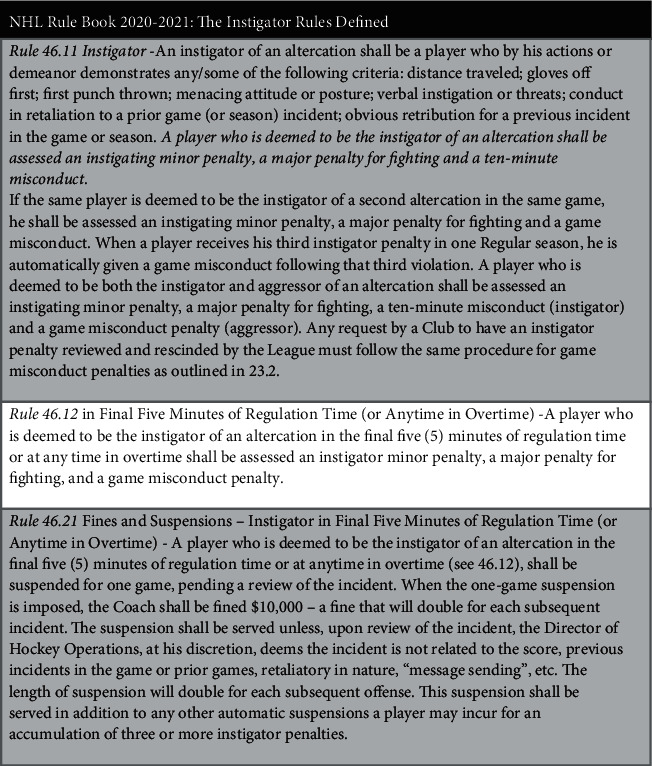
NHL rule book for the 2020–2021 season (under rule 46), with the instigator rule that was initiated during the 1992-1993 season.

In 1992, the instigator rule was introduced which carried harsher punishments for the individual responsible for initiating the fight (Figure 1) [2]. By that time, overall trends in fighting had begun to shift as gameplay became more focused on skill. Furthermore, contemporary data on concussions continued to reshape the relationship between fighting and dangerous gameplay within the NHL [11–13]. Given the negative outcomes associated with concussions and the role fighting plays in increasing concussion rates, it is unsurprising that lawsuits such as Boogaard vs. NHL in 2017 have attempted to address the culture of concussions in hockey [14]. In recent years, committees such as the “Ice Hockey Summit: Action on Concussion” have provided new insights and evidence-based proposals to prevent injuries in the NHL [8]. As such, one topic to come under scrutiny was fighting.

Given the shifting support for fighting, the historical rhetoric of its importance, and the more recent data of harmful health consequences, this paper aims to characterize fighting trends in the NHL. To achieve this goal, we will first characterize the trajectory of fighting rates, then determine the impact of the instigator rule on fighting rates, and finally examine the relationship between fighting and team success. We propose that the rate of fighting has continued to trend downwards, especially following the implementation of the instigator rule, and that increased fighting does not correlate with improved outcomes. Overall, we hope to obtain evidence that fighting provides no significant positive impact in the NHL and that the potential health hazards outweigh the rewards.

## 2. Methods

### 2.1. Data Collection

Statistical data for all NHL teams between 1967 and 2019 were collected from NHL.com. Specific metrics of focus included the season of play, the team record, total penalty minutes, penalty minutes per game, total penalties drawn, total penalties taken, net penalties, net penalties per 60 minutes, penalties drawn and taken per 60 minutes, bench minor penalties, total major penalties, total minor penalties, match penalties, total misconduct penalties, number of game misconducts, points earned, and final standing (i.e., nonplayoff team, playoff team, conference champion, and Stanley Cup champion). Of note, minor penalty minutes do not include fighting minutes in their overall sum. The four outcome categories were defined as follows: nonplayoff teams (teams who failed to make the playoffs), playoff teams (all teams who made the playoffs but advanced no further), conference champions (all teams who won the conference championship but advanced no further), and Stanley Cup champions (teams who won the season championship). For every season, each team was categorized into one of these four categories.

The data were also divided into subgroups according to “era of play.” The 1967 to 1981 seasons were classified as the “Expansion Era,” while the 2005 to 2019 seasons were classified as the “Modern Era.” These two time periods were intentionally chosen as they represent periods in which fighting was most accepted and when fighting was most contentious, respectively. A separate analysis also compared the data before and after the establishment of the instigator rule in 1992. For this analysis, all years were analyzed including 1967 through 2019.

The data were then cross-referenced with another publicly available online database (hockeyfights.com) to obtain data on fighting. Fighting-related data included fights per game and the average time between fights.

### 2.2. Statistical Analysis

Analysis was achieved using the *t*-tests with two-tailed distribution and heteroscedastic variance. Specifically, when comparing the various outcome categories (nonplayoff teams, playoff teams, conference champions, and Stanley Cup champions) between different time eras (i.e., modern vs. expansion) and rule eras (i.e., preinstigator rule vs. postinstigator rule), *t*-tests were used.

Multivariate regression analyses were also conducted with the independent variable being seasonal success (represented as a continuous variable by the number of points achieved that season divided by the number of games played) and the dependent variables being fights/game, net penalty minutes, and minor penalty minutes. Importantly, minor penalty minutes did not incorporate fighting minutes, ensuring variable independence for our testing assumptions. All references to “points” (i.e., points earned) refer to the points earned from wins, overtime wins, and ties, and are not a reference to goals scored.

## 3. Results

**Figure 2:**
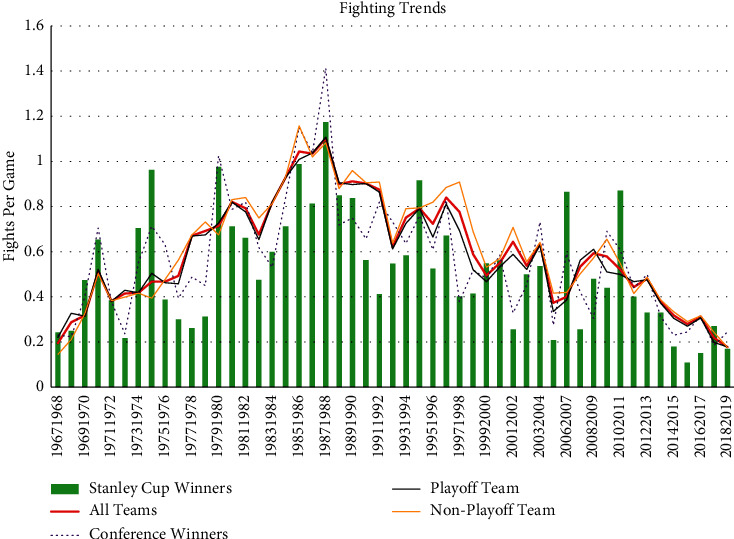
The trends in fights per game from 1967–2019. Fights per game averaged for all teams over each season is depicted by the red line. Green bars represent the average fights per game for the Stanley Cup winner that season. The dotted purple line represents the average fights per game averaged between the 2 conference winning teams that season. The black and yellow lines represent the average number of fights per game averaged between teams who did and did not make the playoffs that season, respectively.

The average number of fights per game has been decreasing since its peak in the 1987 season (Figure 2). When comparing the Expansion Era to the Modern Era, fighting has decreased for both playoff and nonplayoff teams (Table 1). Additionally, there have been significant reductions in the number of penalties per game (all *p* < 0.002) in the Modern Era for teams with all categories of seasonal outcomes.

The instigator rule appears to have reduced the number of fights per game. Fighting decreased from 0.71 per game to 0.51 per game following the implementation of the instigator rule (*p* < 0.0001) (Table 2). For teams with all categories of seasonal outcomes (Stanley Cup winners, conference champions, playoff teams, and nonplayoff teams), the number of fights per game decreased following the implementation of the instigator rule (Table 2). Additionally, total penalty minutes decreased for all categories of teams following the rule change, whereas total penalty minutes increased for nonplayoff teams (Table 2). Furthermore, minor penalty minutes per game decreased following the implementation of the instigator rule (pre = 5.4 ± 1.4 min per game and post = 4.7 ± 1.2 min per game, *p* < 0.001).

**Figure 3:**
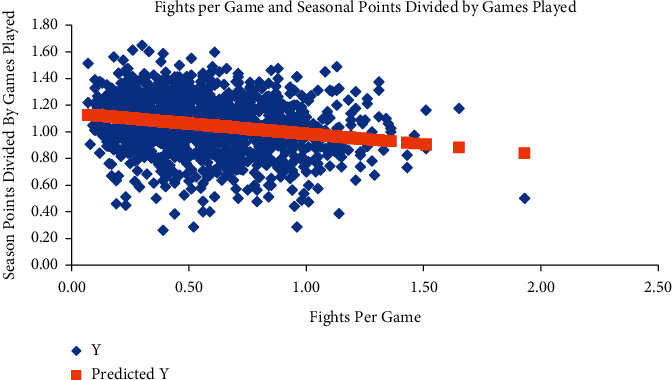
Regression model examining fights per game and seasonal success (measured by total points achieved per season divided by games played). When examining all teams over all seasons, a significant and inverse relationship existed between fighting and the seasonal points earned per game (coefficient = −0.16, *P* < 0.001, *R*^2^ = 0.04). Each blue diamond represents one team during one season.

A regression analysis comparing fights per game and points earned per season divided by the number of games played revealed a statistically significant inverse relationship (coefficient = -0.16, *p* < 0.001) (Figure 3). When a multivariate regression analysis was conducted to control for other penalty types (such as minor penalties, major penalties, and fights), minor penalties had no statistically significant impact on the number of points earned, while fighting maintained its significant negative impact on the number of points earned (coefficient = -0.17, *p* < 0.001).

No significant difference in fighting rates was observed between teams who won the Stanley Cup championship and teams who failed to make the playoffs in either the Modern or Expansion Era (*p* > 0.05) (Table 3). When comparing playoff and nonplayoff teams in both the Modern and Expansion Eras, there were also no differences in fighting rates (*p* > 0.05) (Table 4).

## 4. Discussion

This study has revealed that fighting in the NHL has declined over the recent years and has been significantly influenced by rule modifications such as the instigator rule. Furthermore, there is no observed association between fights per game and seasonal outcomes, despite more fights per game having a statistically significant negative impact on points earned per season. These findings refute existing rhetoric that fighting is helpful in generating more wins per season. These findings may prompt new discussions regarding the role of fighting in the NHL.

Historically speaking, experts have always offered several rationales for keeping fighting in NHL gameplay (Table 5) [6]. One reason frequently mentioned is that fighting acts as a natural deterrent for other dangerous actions on the ice such as slashing, tripping, checking from behind, or other physical altercations. Advocates of fighting state that, without fighting, gameplay would become more dangerous as these alternative actions would become more frequent [6]. Our data, however, suggests this is not the case. Over the past 30 years, we observed that the rate of fighting has continued to decrease. Importantly, when comparing the Expansion Era and the Modern Era, we also see a concomitant decrease in minor penalty minutes per game on average. Thus, not only has the number of fights per game decreased, but so have the other physical altercations that draw minor penalties. This evidence is critical in refuting the notion that fighting is required to curtail other dangerous on ice actions, as we have observed that both fighting and minor penalties have decreased in parallel.

While the implementation of new rules can occasionally have undesired or unforeseen consequences, our analysis clearly demonstrates that the instigator rule has had its intended effect of reducing the frequency of fighting. Initially, the instigator rule carried significant controversy. [15] Despite the initial skepticism, we observed that the instigator rule was successful at reducing not only fighting but the number of minor penalties as well. This finding is critical as it establishes a precedent for new rules having tangible and positive impacts on gameplay and safety. Extrapolating on this finding, it would stand to reason that future rules intended to further reduce or eliminate fighting could have similarly positive impacts, ultimately creating a safer gameplay environment. Of note, the mindset of athletes entering the NHL can vary depending on the league from which they are departing. While the NCAA's tolerance for fighting is nonexistent, players entering the NHL from the Junior Leagues experience a different fighting culture and may be more inclined towards fighting [8]. Both rule changes and a shift in fighting culture would be necessary to fully alter a player's sentiment towards fighting. Lastly, while there are other rules that have had tangible impacts on NHL gameplay (i.e., rule 48 regulating hits to the head), this paper's intended focus on fighting does not incorporate an exhaustive analysis of all rule changes as they are outside the scope of this study.

The final primary goal of this study was to determine if fighting correlated with improved seasonal outcomes. We observed no relationship between the number of fights a team participated in and the ultimate outcome for that team's season. This finding is in line with prior studies that found fighting had no impact, or a slightly negative impact, on a team's success [9]. We observed that not only has fighting decreased when comparing the Expansion Era and the Modern Era, but that fighting has never been a consistent tactic for championship teams. Furthermore, when ignoring the categorical seasonal outcomes (i.e., playoff teams vs. nonplayoff teams) and examining the total points a team earned in any given season based on their wins, overtime wins, and ties, we observed that fighting had a significant negative impact on points earned per season (Figure 3). An additional multivariate regression model controlling for other factors that could impact team success was also created, and it too concluded that fighting had a statistically significant negative impact on points earned. This further strengthens the notion that fighting not only fails to increase seasonal success, but it may actually be detrimental.

With respect to injury prevention, the presence of fighting is exclusively detrimental to player safety. A comprehensive review of concussions concluded that fighting alone accounted for 9% of all concussions in the NHL [16,17]. Outside of concussions, players participating in fights are susceptible to other injuries such as wrist/finger/orbital fractures and broken nose that can render them unable to play for numerous games. It was previously mentioned that fighting provided a source for increased ticket sales and, therefore, increased revenue. While this may be true, it fails to consider the downstream consequences of fights [10]. Direct costs associated with fighting, which offset the gains realized by increased ticket sales include direct medical treatment costs, insurance costs, and most significantly, contract losses when players are injured. NHL teams sign significant monetary contracts with players, and when those players are injured in fights and can no longer participate, the portion of their contract that correlates with their absence is functionally rendered a financial loss. Furthermore, when star players are injured, their absence can have an equally drastic but negative impact on fan attendance and ticket sales. The economic burden of NHL injuries is measured to be $218 million annually [18].

An important takeaway arises when we consider these findings together. First, fighting has been declining over the past 30 years, and so has the number of minor penalties, suggesting fighting is not necessary to curtail alternative violent rule infarctions. Second, in both the Modern Era and the Expansion Era (the era known to be more sympathetic towards fighting), we see no evidence that fighting has a positive impact on winning. Finally, when examining the impact of the instigator rule (a rule intended to curtail fighting), we indeed observed a reduction in the number of fights during gameplay. Considering these findings together and the recent literature on injuries and player safety, we see compelling evidence that a further reduction in NHL fighting would be unlikely to impact the NHL in a negative manner.

Limitations of this study include its retrospective design and the limitation of the data available for analysis. Despite the majority of the data coming from the official NHL source, it is indeed a public site with the potential for occasional discrepancies. We attempted to cross-reference the data as frequently as possible with other publicly available records to mitigate this potential error. Using NHL gameplay footage to validate all data, however, was logistically unfeasible and remains a limitation. Furthermore, the focus of this paper is the impact of fighting at the team level for seasonal success and at the player level for injuries. Alternative motivations for fighting such as respect earned by other players, increased playing time, roster positions, desire to protect skilled players, and contract extensions are difficult to evaluate given the challenge of ascertaining empirical data, and thus are not included in this analysis. Therefore, the focus for this study remained at the team level and for the NHL as an organization.

## 5. Conclusion

Fighting in the NHL has been consistently declining over the past 30 years. This study observed that fighting was never found to consistently improve team success and that fighting was in fact detrimental. Furthermore, recent rule changes to reduce fighting (i.e., the instigator rule) have been successfully implemented with no offsetting rise in minor penalties. These findings, especially when considered in the context of recent player injury data, provide evidence to reexamine the relationship between fighting and the NHL and consider the appropriate trajectory moving forward.

## Figures and Tables

**Table 1:** Fighting and penalty data comparing the Modern Era and the Expansion Era.

Fights/gm			Total penalty (minutes/gm)			Net penalty (mins/gm)		
Mean	SD	*p* value	Mean	SD	*p* value	Mean	SD	*p* value
Stanley Cup champions	2005–2019	0.36	0.24	0.24	11.01	2.90	0.002	0.17	0.36	0.002
	1967–1980	0.45	0.21		14.50	3.75		−0.41	0.77	
Conference champions	2005–2019	0.38	0.23	0.10	11.24	2.75	<0.0001	0.13	0.36	0.002
	1967–1980	0.47	0.21		15.18	3.67		−0.38	0.78	
Playoff teams	2005–2019	0.40	0.20	<0.0001	11.18	2.61	<0.0001	0.02	0.37	<0.0001
	1967–1980	0.51	0.23		14.40	4.11		−0.03	0.68	
Nonplayoff teams	2005–2019	0.42	0.19	<0.0001	12.06	3.06	<0.0001	−0.04	0.47	0.02

**Table 2:** The impact of the instigator rule on the NHL.

Fights/gm			Total penalty (minutes/gm)			Net penalty (mins/gm)		
Mean	SD	*p* value	Mean	SD	*p* value	Mean	SD	*p* value
Stanley Cup champions	Preinstigator rule	0.60	0.27	0.03	13.00	1.88	0.01	−0.43	0.79	0.001
	Postinstigator rule	0.44	0.22		10.44	1.32		0.18	0.37	
Conference champions	Preinstigator rule	0.66	0.33	0.002	13.26	1.89	0.0002	−0.38	0.78	<0.0001
	Postinstigator rule	0.48	0.23		10.74	1.43		0.15	0.40	
Playoff teams	Preinstigator rule	0.73	0.31	<0.0001	13.46	1.73	<0.0001	−0.08	0.63	0.0002
	Postinstigator rule	0.50	0.23		10.92	1.42		0.07	0.43	
Nonplayoff teams	Preinstigator rule	0.67	0.31	<0.0001	10.90	1.42	<0.0001	0.20	0.56	<0.0001
	Postinstigator rule	0.53	0.24		12.30	1.74		−0.09	0.45	
Average over all teams:										
	Fights/game	SD	*p* value							
Preinstigator rule	0.71	0.31	<0.0001							
Postinstigator rule	0.51	0.24								

**Table 3:** Fighting and penalty data comparing NHL Stanley Cup champions and nonplayoff teams.

	Stanley Cup champions		Nonplayoff teams		*p*value
	Mean	SD	Mean	SD	
Modern Era (2005–2019)					
Fights/gm	0.36	0.24	0.42	0.19	0.44
Total penalty (minutes/gm)	11.01	2.90	12.06	3.06	0.15
Net penalty (mins/gm)	0.17	0.36	-0.04	0.47	0.03
Expansion Era (1967–1980)					
Fights/gm	0.45	0.21	0.51	0.24	0.49
Total penalty (minutes/gm)	14.50	3.75	13.74	3.61	0.01
Net penalty (mins/gm)	-0.41	0.77	0.08	0.62	<0.0001

Gm = game.

**Table 4:** Fights and penalty data comparing playoff teams and nonplayoff teams.

	Playoff teams		Nonplayoff teams		*p*value
	Mean	SD	Mean	SD	
Modern Era (2005−2019)					
Fights/gm	0.40	0.20	0.42	0.19	0.46
Total penalty (minutes/gm)	11.18	2.61	12.06	3.06	0.002
Net penalty (mins/gm)	0.02	0.37	-0.04	0.47	0.03
Expansion Era (1967−1980)					
Fights/gm	0.51	0.23	0.51	0.24	0.33
Total penalty (minutes/gm)	14.40	4.11	13.74	3.61	0.0007
Net penalty (mins/gm)	−0.03	0.68	0.08	0.62	<0.0001

Gm = game.

**Table 5:** Reasons offered to continue to allow fighting in the National Hockey League.

Reasons given to continue to allow fighting in professional hockey
Able to change momentum of the gameplay
Enabling players to self-regulate other, lesser, violent gameplay
Intimidation style of hockey (Broad Street Bullies)
Helps win games
Increases ticket sales
Protects the star players and enables them to utilize their skillsets

[6]

## Data Availability

The majority of the data was collected from the official NHL statistics warehouse. http://www.nhl.com/stats/ Fighting-specific data was also obtained from the primary NHL fighting collection site (https://www.hockeyfights.com).
